# Supplementary material for: Identification of Regulatory Molecular “Hot Spots” for LH/PLOD Collagen Glycosyltransferase Activity
Source: Int J Mol Sci. 2023 Jul 7;24(13):11213. doi: 10.3390/ijms241311213 (PMC10342707; doi:10.3390/ijms241311213)
Supplement: Supplementary file 1 [file ijms-24-11213-s001.zip › ijms-2352294-supplementary.pdf]

Supplementary Data for

Identification of regulatory molecular hot spots for LH/PLOD collagen glycosyltransferase activity

D. Mattoteia, A. Chiapparino, *et al.*

Table S1: crystallographic statistics for data collection, structure solution and refinement.

[a] Values in parentheses are for reflections in the highest resolution shell.

	LH3/PLOD3<br>+ Fe^{2+} + Mn^{2+}<br>+ UDP	LH3/PLOD3<br>+ Fe^{2+} + Mn^{2+}<br>+ UDP-GlcA	LH3/PLOD3<br>+ Fe^{2+} + Mn^{2+}<br>+ UDP-Xyl	LH3/PLOD3<br>Val80Lys<br>+ Fe^{2+} + Mn^{2+}	LH3/PLOD3<br>Val80Lys<br>+ Fe^{2+} + Mn^{2+}<br>+ UDP-Glc	LH3/PLOD3<br>Val80Lys<br>+ Fe^{2+} + Mn^{2+}<br>+ UDP-GlcA	LH3/PLOD3<br>Asp190Ser + Fe^{2+}<br>+ Mn^{2+}<br>+ UDP-Glc
**Data Collection[a]**							
X-ray source	ESRF ID30A-3	SLS X06SA	ESRF ID23-EH2	SLS X06SA	SLS X06SA	SLS X06SA	ESRF ID23-EH2
Processing programs	XDS, AIMLESS, STARANISO	XDS, AIMLESS, STARANISO	XDS, AIMLESS, STARANISO	XDS, AIMLESS, STARANISO	XDS, AIMLESS, STARANISO	XDS, AIMLESS, STARANISO	XDS, AIMLESS, STARANISO
Space group	C222$_1$	C222$_1$	C222$_1$	C222$_1$	C222$_1$	C222$_1$	C222$_1$
Cell parameters	a = 97.0 Å; α = 90°<br>b = 100.0 Å; β = 90°<br>c = 225.2 Å; γ = 90°	a = 98.2 Å; α = 90°<br>b = 100.5 Å; β = 90°<br>c = 224.7 Å; γ = 90°	a = 97.2 Å; α = 90°<br>b = 100.0 Å; β = 90°<br>c = 224.0 Å; γ = 90°	a = 98.0 Å; α = 90°<br>b = 100.8 Å; β = 90°<br>c = 225.7 Å; γ = 90°	a = 98.1 Å; α = 90°<br>b = 100.4 Å; β = 90°<br>c = 225.2 Å; γ = 90°	a = 98.0 Å; α = 90°<br>b = 99.8 Å; β = 90°<br>c = 224.5 Å; γ = 90°	a = 97.1 Å; α = 90°<br>b = 100.2 Å; β = 90°<br>c = 223.8 Å; γ = 90°
Wavelength (Å)	0.968	1.000	0.873	1.000	1.000	1.000	0.775
Resolution (Å)	48.84-2.30<br>(2.38-2.30)	49.10-2.20<br>(2.26-2.20)	48.79-2.40<br>(2.49-2.40)	49.14-3.00<br>(3.18-3.00)	49.02-2.30<br>(2.38-2.30)	49.00-2.70<br>(2.83-2.70)	48.88-2.30<br>(2.38-2.30)
Total reflections	230588 (21551)	430059 (32322)	237715 (25682)	93694 (15275)	251674 (15330)	197473 (22212)	322199 (25283)
Unique reflections	48288 (4455)	56604 (4537)	43005 (4500)	22529 (3578)	49068 (4243)	30477 (3911)	48804 (4443)
CC1/2[b]	0.997 (0.512)	0.999 (0.860)	0.992 (0.586)	0.986 (0.490)	0.998 (0.461)	0.996 (0.580)	0.995 (0.587)
Redundancy	4.8 (4.8)	7.6 (7.1)	5.5 (5.7)	4.2 (4.3)	5.1 (3.6)	6.5 (5.7)	6.6 (5.7)
Mean I/σ(I)	8.4 (0.9)	11.8 (0.5)	5.5 (0.7)	4.5 (0.7)	6.7 (0.8)	9.4 (1.3)	5.7 (0.5)
Completeness (%)	98.7 (99.7)	99.8 (98.6)	99.9 (99.9)	99.0 (98.5)	98.7 (94.0)	99.5 (98.3)	99.9 (99.9)
R_{sym}[b]	0.118 (1.476)	0.104 (2.566)	0.209 (1.831)	0.227 (3.045)	0.100 (1.351)	0.138 (1.312)	0.175 (1.827)
R_{pim}[c]	0.086 (1.107)	0.060 (1.554)	0.147 (1.274)	0.186 (2.479)	0.071 (1.155)	0.088 (0.902)	0.107 (1.255)

[b] $R_{sym} = [\Sigma_{hkl}\Sigma_j \mid I_{hkl,j} - \langle I_{hkl}\rangle \mid] / [\Sigma_{hkl}\Sigma_j I_{hkl,j}]$, where I is the observed intensity for a reflection and <I> is the average intensity obtained from multiple observations of symmetry-related reflections.

[c] $R_{pim} = [\Sigma_{hkl} (1/(n-1))^{1/2} \Sigma_j \mid I_{hkl,j} - \langle I_{hkl}\rangle \mid] / [\Sigma_{hkl}\Sigma_j I_{hkl,j}]$ where I is the observed intensity for a reflection and <I> is the average intensity obtained from multiple observations of symmetry-related reflections.

	LH3/PLOD3 + Fe^{2+} + Mn^{2+} + UDP	LH3/PLOD3 + Fe^{2+} + Mn^{2+} + UDP-GlcA	LH3/PLOD3 + Fe^{2+} + Mn^{2+} + UDP-Xyl	LH3/PLOD3 Val80Lys + Fe^{2+} + Mn^{2+}	LH3/PLOD3 Val80Lys + Fe^{2+} + Mn^{2+} + UDP-Glc	LH3/PLOD3 Val80Lys + Fe^{2+} + Mn^{2+} + UDP-GlcA	LH3/PLOD3 Asp190Ser + Fe^{2+} + Mn^{2+} + UDP-Glc
**Refinement**							
R_{work}/R_{free}[c]	0.1943/0.2458	0.1982/0.2411	0.1852/0.2159	0.2079/0.2412	0.1701/0.2268	0.1895/0.2278	0.2043/0.2288
Number of atoms:	5874	6293	6019	5732	5938	5893	6125
Protein	5646	5754	5754	5665	5736	5716	5693
Ligands	73	100	105	67	92	104	109
Solvent	155	439	160	-	110	73	323
Average B-factor (Å)^2	35.83	36.51	31.45	42.33	44.80	43.73	35.71
Protein	35.73	35.91	31.16	42.07	44.54	42.99	35.33
Ligands	56.66	58.25	53.94	63.99	64.67	91.24	54.65
Solvent	29.78	39.40	26.84	-	41.54	33.94	36.11
**Structure quality**							
RMS bond lengths (Å)	0.006	0.005	0.003	0.004	0.009	0.003	0.002
RMS bond angles (°)	0.78	0.84	0.58	0.70	1.05	0.71	0.50
**Ramachandran stats**							
Favored (%)	97.5	97.1	97.0	95.9	96.3	96.5	96.5
allowed (%)	2.5	2.7	2.9	3.8	3.6	3.3	3.0
outliers (%)	0.0	0.2	0.1	0.3	0.1	0.2	0.5
PDB ID	6TE3	6TES	6TEC	6TEU	6TEX	6TEZ	8ONE

[c] R_{free} values are calculated based on 5% randomly selected reflections, selected prior to STARANISO correction as recommended by the software developers (Tickle et al, 2018).

Table S2: List of glycosyltransferase enzymes used for comparisons with human LH3/PLOD3. The list includes the indication of the catalytic bases and nucleophile residues as proposed in the original papers describing the various glycosyltransferases, with the corresponding residue number in human LH3/PLOD3 based on structural superpositions.

Protein Name	Type	PDB ID	catalytic base residue	nucleophile acceptor residue	corresp. LH3/PLOD3 residue	reference paper
LgtC - GALACTOSYL TRANSFERASE LGTC *(N. meningitidis)*	retaining	1GA8				Persson et al., 2001 10.1038/84168
GYG1 - Glycogenin *(O. cuniculus)*	retaining	1LL2		Asp163	Asp191	Gibbons et al., 2002 10.1016/S0022-2836(02)00305-4
mgs - Mannosylglycerate synthase (*R. marinus)*	retaining	2BO8				Flint et al., 2005 10.1038/nsmb950
GALNT10 - Polypeptide N-acetylgalactosaminyl transferase 10 (*H. sapiens)*	retaining	2D7R		Gln346	Asp190	Kubota et al., 2006 10.1016/j.jmb.2006.03.061
GGTA1 - N-Acetyllactosaminide α-1,3-galactosyl transferase (R365K) *(B. taurus)*	retaining	5NRB		Glu317	Gln192	Albesa-Jove et al., 2017 10.1002/anie.201707922
ABO - Histo-blood group ABO system transferase *(H. sapiens)*	retaining	1LZI		Glu303	Gln192	Patenaude et al., 2002 10.1038/nsb832
spsA - PROTEIN (SPORE COAT POLYSACCHARIDE BIOSYNTHESIS PROTEIN SPSA)	inverting	1QGQ	Asp191		Asp191	Charnock et al., 1999 10.1128/JB.183.1.77-85.2001
MGAT1 - N-acetylglucosaminyl transferase I *(O. cuniculus)*	inverting	1FOA	Asp 291		Asp191	Unligil et al., 2000 10.1093/emboj/19.20.5269
Mfng - Manic Fringe glycosyltransferase *(M. musculus)*	inverting	2J0B	Asp 232		Asp191	Jinek et al., 2006 10.1038/nsmb1144
B3GAT3 - GLUCURONYLTRANSFERASE I	inverting	1FGG	Glu281		Asp190	Pedersen et al., 2000 10.1074/jbc.M007399200
B3GAT1 – Galactosylgalactosylxylosyl protein 3-beta-glucuronosyltransferase 1		1V84				Kakuda et al., 2004 10.1074/jbc.M400622200

Table S3: list of oligonucleotides used for mutagenesis

Oligonucleotide Name	Sequence (5'→3')
Forward LH3/PLOD3 Val80Lys	AAGGCTCGAACAGTTGGTGGAGGAC
Reverse LH3/PLOD3 Val80Lys	ATCACCCCCTCGCCACTCCTC
Forward LH3/PLOD3 Val80Gly	GAGCTCGAACAGTTGGTGGAGGAC
Reverse LH3/PLOD3 Val80Gly	CATCACCCCCTCGCCACTCC
Forward LH3/PLOD3 Trp92Ala	GCATTAAAGAAGGAAATGGAGAAATACG
Reverse LH3/PLOD3 Trp92Ala	CCGGACCTTCTGTCCTCCACC
Forward LH3/PLOD3 Glu141Ala	CGAGCTTCTGCTGGCCCGAGTG
Reverse LH3/PLOD3 Glu141Ala	CTGCAGAGAAGAGCAGGCGGCTG
Forward LH3/PLOD3 Trp145Ala	GCACCCGAGTGGGGGCTGGC
Reverse LH3/PLOD3 Trp145Ala	GCAGAAGCTCTCTGCAGAGAAGAGC
Forward LH3/PLOD3 Trp148Ala	GCAGGGCTGGCGGAGCAGTAC
Reverse LH3/PLOD3 Trp148Ala	CTCGGGCCAGCAGAAGCTCTC
Forward LH3/PLOD3 Asn165Ala	GCTTCTGGTGGATTCATCGGTTTTGC
Reverse LH3/PLOD3 Asn165Ala	GAGGAAGCGCTTCCCCGTGC
Forward LH3/PLOD3 Asp190Ala	CTGACCAGCTGTTCTACACACGGC
Reverse LH3/PLOD3 Asp190Ala	CGTCATCATCCTTGTACTTCCACTGG
Forward LH3/PLOD3 Asp190Ser	AGCGACCAGCTGTTCTACACACGGCT
Reverse LH3/PLOD3 Asp190Ser	GTCATCATCCTTGTACTTCCACTGGCG
Forward LH3/PLOD3 Asp191Ala	CTCAGCTGTTCTACACACGGCTC
Reverse LH3/PLOD3 Asp191Ala	CGTCGTCATCATCCTTGTACTTCCAC
Forward LH3/PLOD3 Gln192Ala	GCGCTGTTCTACACACGGCTCTAC
Reverse LH3/PLOD3 Gln192Ala	GTCGTCGTCATCATCCTTGTACTTCC
Forward LH3/PLOD3 Asn255Ala	GCCGGTCCCACTAAGCTGCAGC
Reverse LH3/PLOD3 Asn255Ala	TCCATGGACCACAATGGGGAGCGTG
Forward LH3/PLOD3 Pro270Leu	TCAATGGCTGGACTCCTGAGGG
Reverse LH3/PLOD3 Pro270Leu	GGACGTAGTTTCCCAGGTAGTTGAG
Forward LH1/PLOD1 Ser178Asp	GACGATCAGCTGTTTTACACCAAGATC
Reverse LH1/PLOD1 Ser178Asp	GTCGCTGTCCTGGCCCTCCCACTCGG

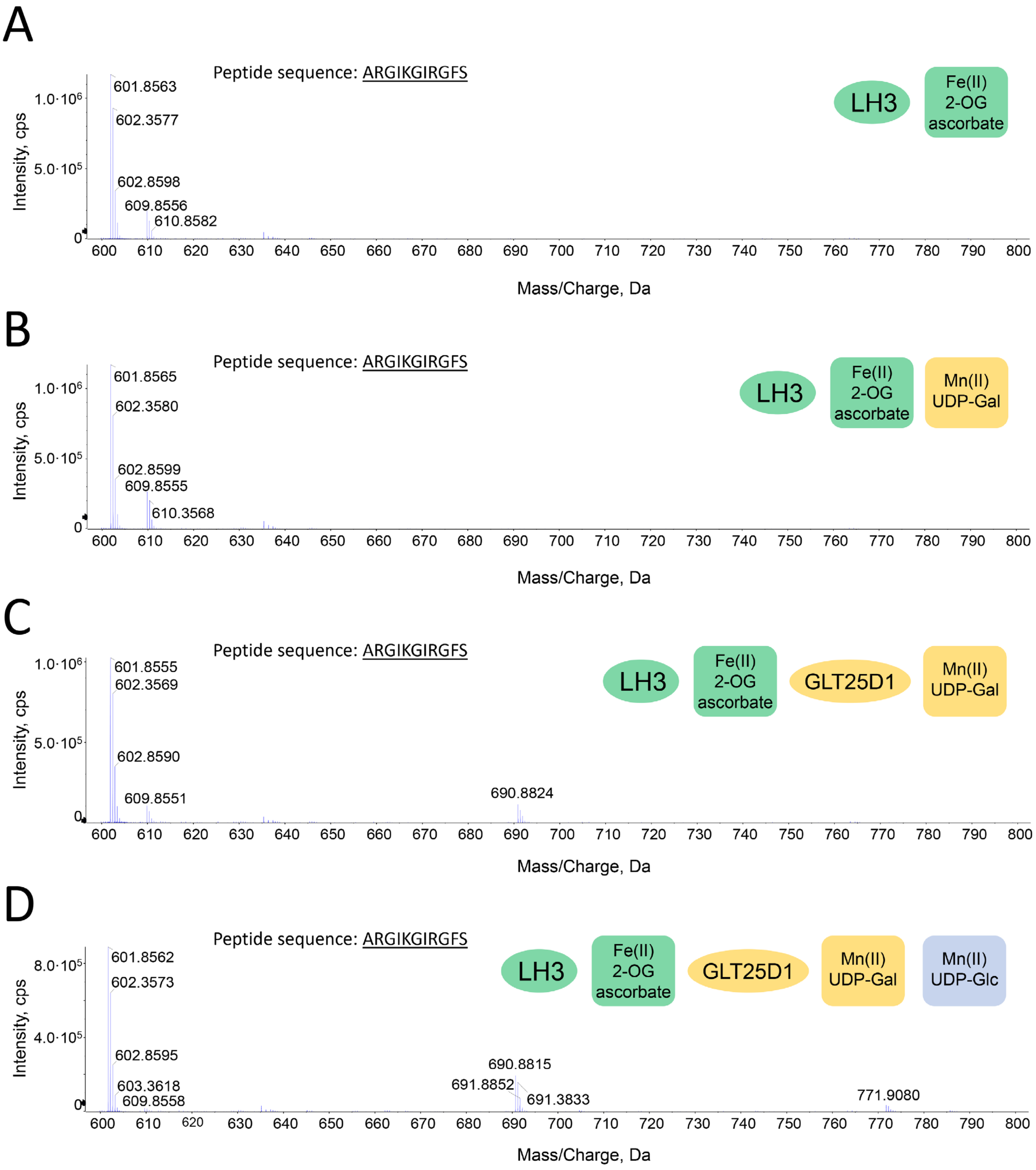
A
Peptide sequence: ARGIKGIRGFS
LH3
Fe(II)
2-OG
ascorbate
Intensity, cps
Mass/Charge, Da
601.8563
602.3577
602.8598
609.8556
610.8582
B
Peptide sequence: ARGIKGIRGFS
LH3
Fe(II)
2-OG
ascorbate
Mn(II)
UDP-Gal
601.8565
602.3580
602.8599
609.8555
610.3568
C
Peptide sequence: ARGIKGIRGFS
LH3
Fe(II)
2-OG
ascorbate
GLT25D1
Mn(II)
UDP-Gal
601.8555
602.3569
602.8590
609.8551
690.8824
D
Peptide sequence: ARGIKGIRGFS
LH3
Fe(II)
2-OG
ascorbate
GLT25D1
Mn(II)
UDP-Gal
Mn(II)
UDP-Glc
601.8562
602.3573
602.8595
603.3618
609.8558
690.8815
691.8852
691.3833
771.9080

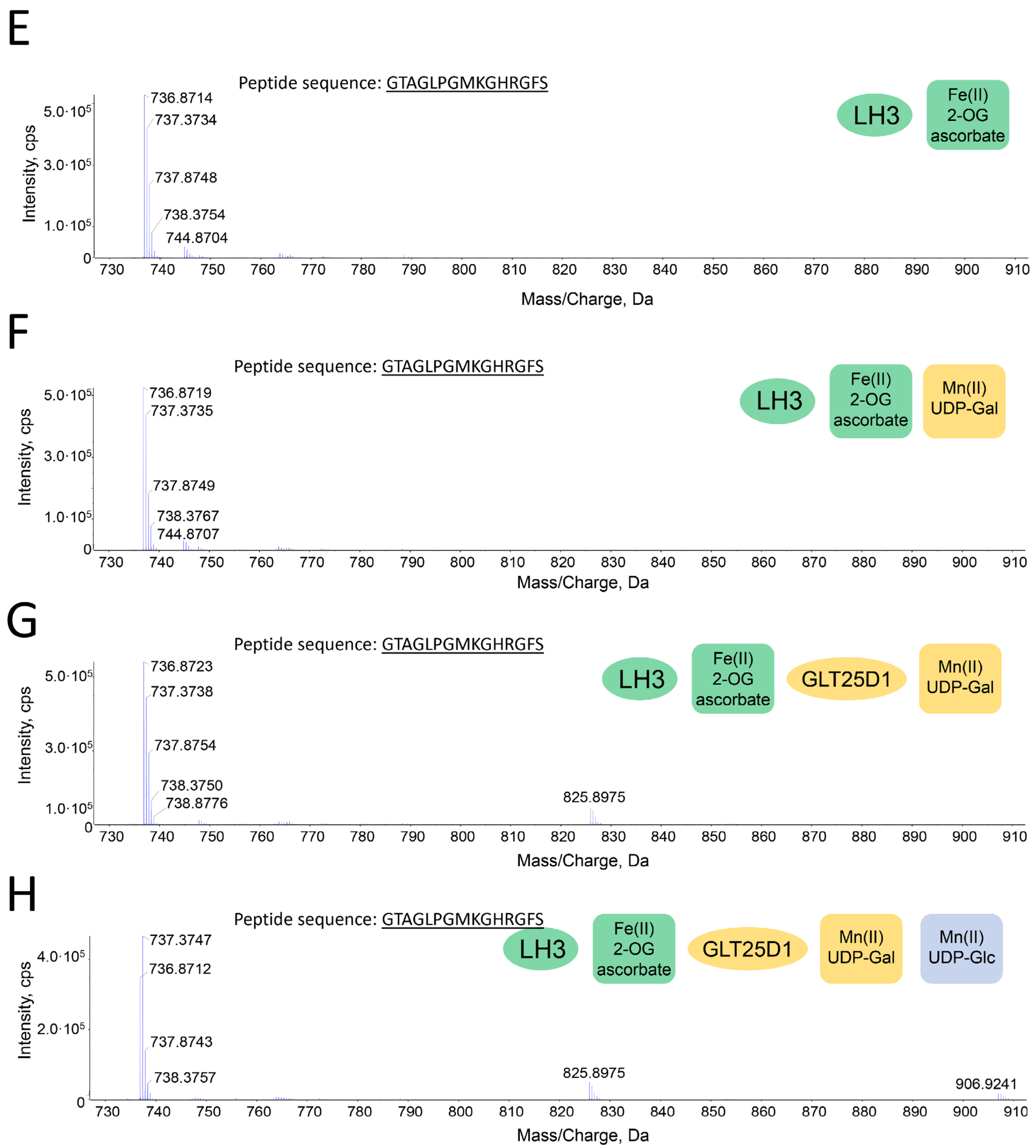

Figure S1: Results of the direct MS activity assays with two different synthetic peptides other than GIKGIKGIKGIK shown in figure 1. Panels (A) to (D): peptide sequence ARGIKGIRGFS; panels (E) to (H): peptide sequence GTAGLPGMKGHRGFS.

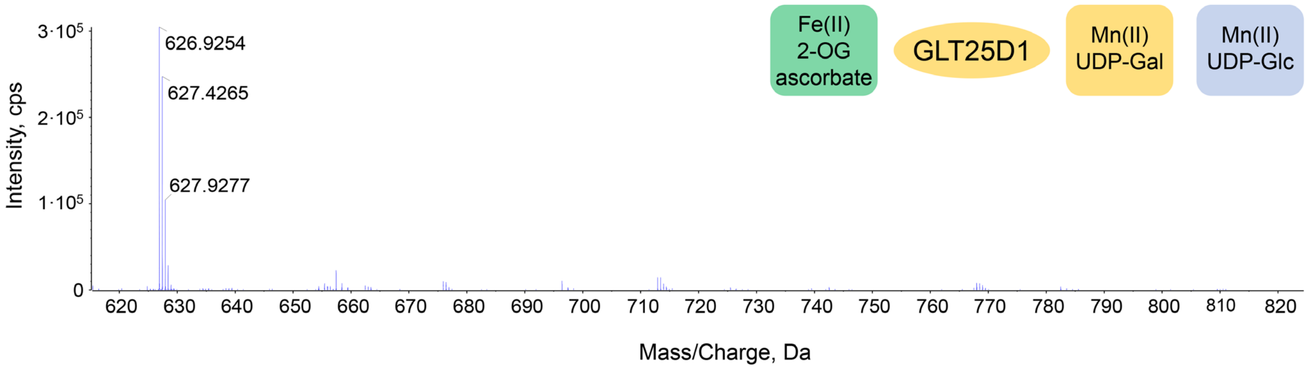

Figure S2: Direct MS activity assay to evaluate Lys-to-Hyl conversion in absence of LH/PLOD enzymes. The experiment shown was performed using the GIKGIKGIKGIK peptide. Results exclusively show the peak corresponding to the unmodified peptide (627 Da, doubly charged).

A

Glycoloop

```
                 33        43         53         63         73         83
Human LH1/PLOD1 AKPEDNLLVL TVATKETEGF RRFKRSAQFF NYKIQALGLG EDWN-VEKGT SAGGGQKVRL
Human LH2/PLOD2 SIPTDKLLVI TVATKESDGF HRFMQSAKYF NYTVKVLGQG EEWRGGDGIN SIGGGQKVRL
Human LH3/PLOD3 PVNPEKLLVI TVATAETEGY LRFLRSAEFF NYTVRTLGLG EEWRGGDVAR TVGGGQKVRW
consensus        .   ::***: **** *::*:   ** .**::* **:.:.** * *:*.  .     : *******

                 93        103        113        123        133        143
Human LH1/PLOD1 LKKALEKHAD KEDLVILFAD SYDVLFASGP RELLKKFRQA RSQVVFSAEE LIYPDRRLET
Human LH2/PLOD2 MKEVMEHYAD QDDLVVMFTE CFDVIFAGGP EEVLKKFQKA NHKVVFAADG ILWPDKRLAD
Human LH3/PLOD3 LKKEMEKYAD REDMIIMFVD SYDVILAGSP TELLKKFVQS GSRLLFSAES FCWPEWGLAE
consensus        :*: :*::** .:*::::*.: .:**::*..* *:**** ::    .::*:*:   : :*:. *
```

PolyAsp

```
                 153       163        173        183        193        203
Human LH1/PLOD1 KYPVVSDGKR FLGSGGFIGY APNLSKLVAE WEGQDSDSDQ LFYTKIFLDP EKREQINITL
Human LH2/PLOD2 KYPVVHIGKR YLNSGGFIGY APYVNRIVQQ WNLQDNDDDQ LFYTKVYIDP LKREAINITL
Human LH3/PLOD3 QYPEVGTGKR FLNSGGFIGF ATTIHQIVRQ WKYKDDDDDQ LFYTRLYLDP GLREKLSLNL
consensus        :** *  *** :*.******: *. : .:* : *: :*.*.** ****.:::**   ** :.:.*

                 213       223        233        243        253        263
Human LH1/PLOD1 DHRCRIFQNL DGALDEVVLK FEMGHVRARN LAYDTLPVLI HGNGPTKLQL NYLGNYIPRF
Human LH2/PLOD2 DHKCKIFQTL NGAVDEVVLK FENGKARAKN TFYETLPVAI NGNGPTKILL NYFGNYVPNS
Human LH3/PLOD3 DHKSRIFQNL NGALDEVVLK FDRNRVRIRN VAYDTLPIVV HGNGPTKLQL NYLGNYVPNG
consensus        **...***.* :**:****** *: ...* .*   *:***: : :******: * **:***:*.

                 273       283
Human LH1/PLOD1 WTFETGCTVC DEGLRSLKGI
Human LH2/PLOD2 WTQDNGCTLC EFDTVDLSAV
Human LH3/PLOD3 WTPEGGCGFC NQDRRTLPGG
consensus        ** : ** .* : .   * .
```

B

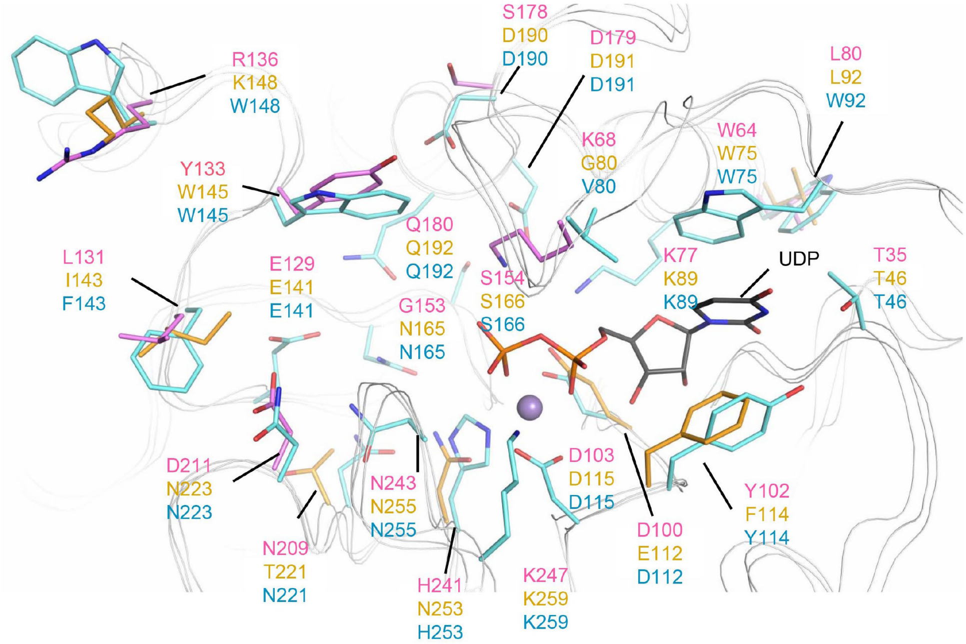

Figure S3: Conserved features of the GT domains in human LH/PLOD isoenzymes. (A) sequence alignment of human LH1/PLOD1, LH2/PLOD2 and LH3/PLOD3 GT domains, highlighting similarities and differences in the amino acid residues within the active site. Residues highlithed in Figure 2A are indicated here with a triangle. Colored boxes indicate the PolyAsp motif (brown) and the Glycoloop (cyan). (B) Superposition of the GT site of LH3/PLOD3 in complex with UDP-glucose (PDB ID: 6FXT) and LH1/PLOD1 and LH2/PLOD2 homology models (Scietti et al, 2019) (available at http://fornerislab.unipv.it/SiMPLOD/). Aminoacids identified as part of the LH3/PLOD3 catalytic site are shown as light blue sticks, while the LH1/PLOD1 and LH2/PLOD2 residues which differs from LH3/PLOD are shown in pink and orange respectively. UDP moiety (black lines) and Mn^{2+} cofactor (purple sphere) are also shown from the LH3/PLOD3 structure.

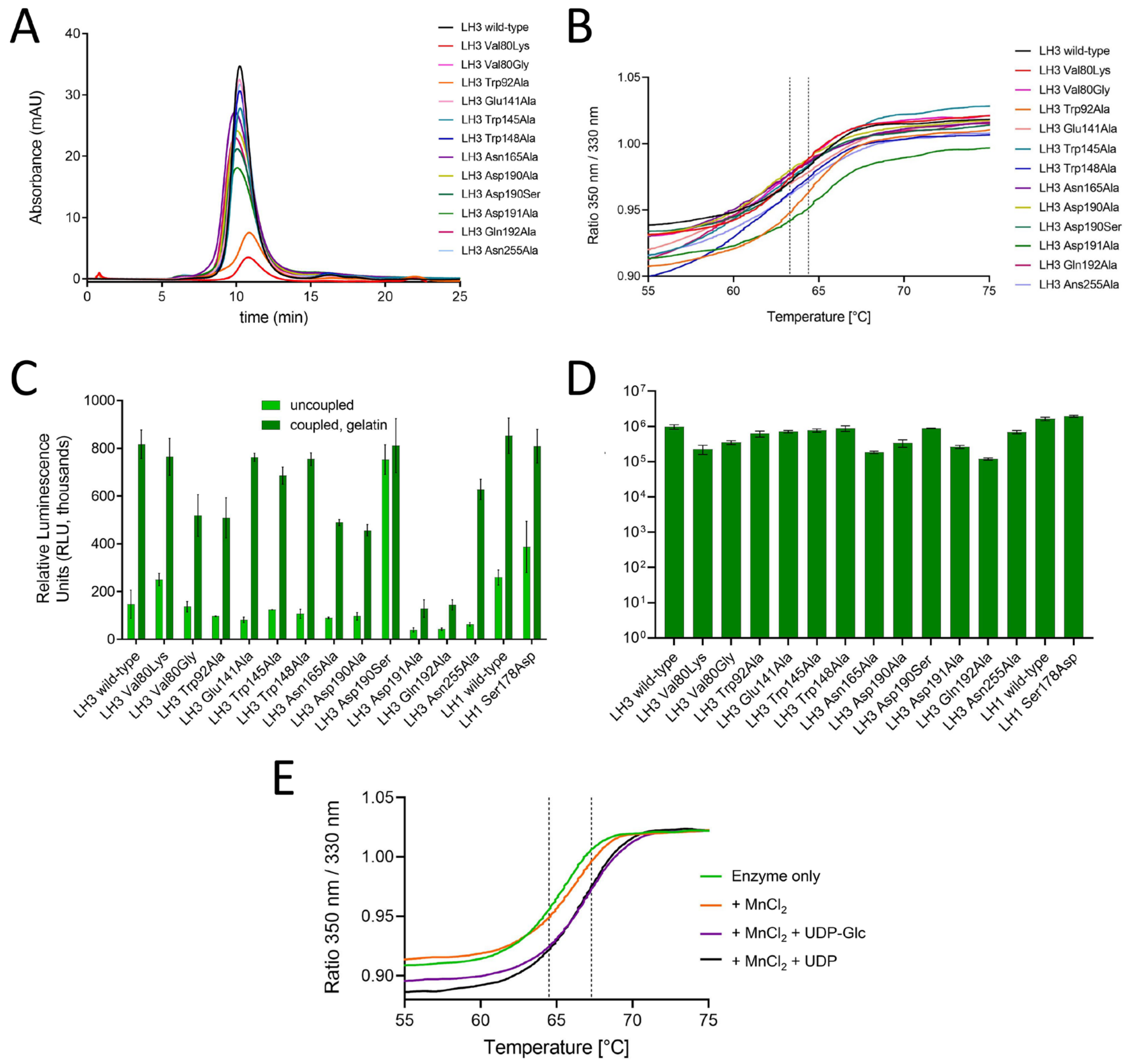

Figure S4: biochemical evaluation of LH3/PLOD3 wild-type and mutants. (A) Analytical size-exclusion chromatography analysis comparing wild-type and mutants LH3/PLOD3. (B) DSF comparing wild-type and mutants LH3/PLOD3. The dashed lines indicate the temperature range incorporating the calculated T_m values for all curves. (C) LH activity comparison for wild-type and mutants LH3/PLOD3 using indirect luminescence-based assays. The measurements were performed in absence ("uncoupled") and in presence ("coupled") of gelatin acceptor substrates, as described in (Scietti et al, 2018). Error bars represent standard deviations from average of triplicate independent experiments. (D) Assessment of LH enzymatic activity using direct MS-based assays for all LH3/PLOD3 mutants described in this work. (E) DSF analysis of UDP and UDP-Glc binding by LH3/PLOD3 Asp190Ser mutant.

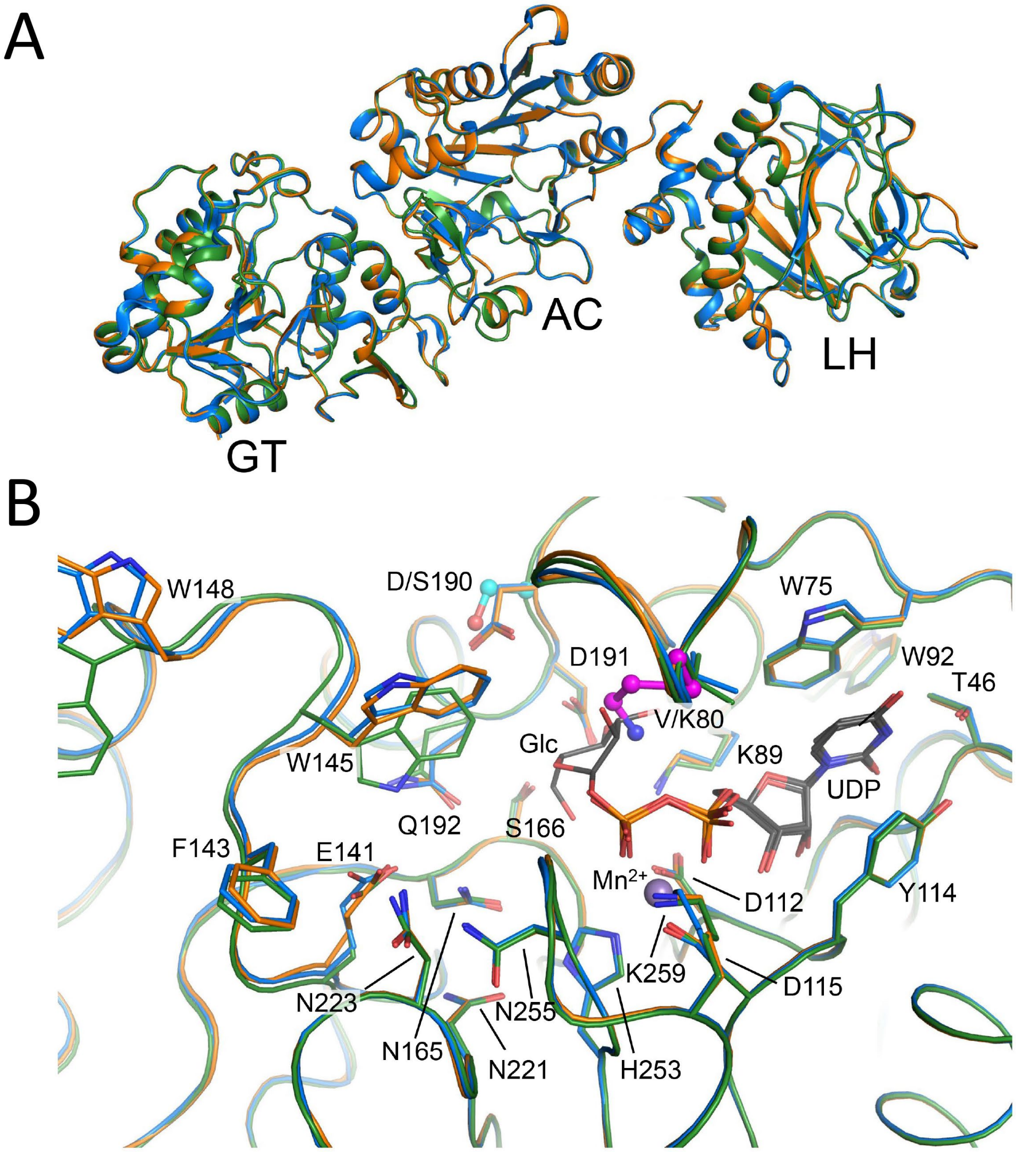

Figure S5: Structural comparison of the LH3/PLOD3 wild-type and mutant structures in complex with UDP-glucose. (A) The overall structure superposition of the asymmetric units of LH3/PLOD3 wild-type (blue), Val80Lys (orange), and Asp190Ser (green) shows that the two structures are almost identical. (B) Zoomed view of the GT active site of the superimposed structures shown in (A) with the same overall coloring, indicating that the sole differences are constituted by the mutated Val80 to Lys in the Val80Lys structure (shown in pink ball-and-stick) and the mutated Asp190 to Ser in the Asp190Ser structure (shown in cyan ball-and stick).

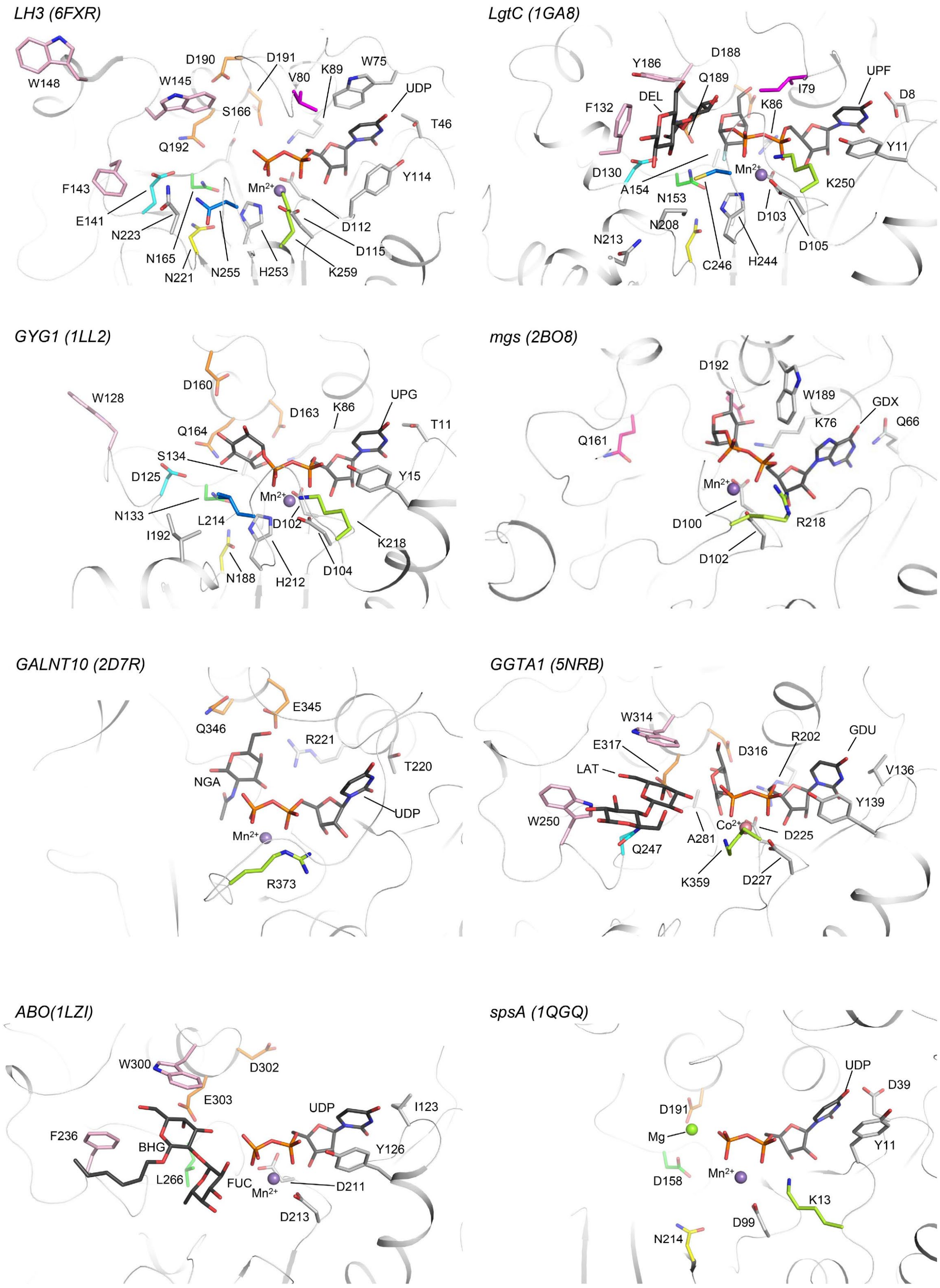
LH3 (6FXR)
W148
D190
D191
V80
K89
W75
W145
S166
UDP
T46
Q192
F143
Mn2+
Y114
E141
N223
N165
N221
N255
H253
K259
D112
D115
LgtC (1GA8)
Y186
D188
Q189
I79
UPF
D8
DEL
F132
K86
Y11
D130
A154
Mn2+
K250
N153
N208
D103
N213
C246
H244
D105
GYG1 (1LL2)
W128
D160
D163
K86
UPG
T11
Q164
S134
D125
Y15
N133
L214
Mn2+
D102
K218
I192
N188
H212
D104
mgs (2BO8)
D192
W189
GDX
Q66
K76
Q161
Mn2+
D100
R218
D102
GALNT10 (2D7R)
E345
Q346
R221
T220
NGA
UDP
Mn2+
R373
GGTA1 (5NRB)
W314
E317
D316
R202
GDU
V136
LAT
Y139
W250
A281
Co2+
D225
Q247
K359
D227
ABO(1LZI)
W300
D302
E303
UDP
I123
F236
BHG
Y126
L266
FUC
Mn2+
D211
D213
spsA (1QGQ)
UDP
D39
D191
Mg
Y11
D158
Mn2+
K13
D99
N214

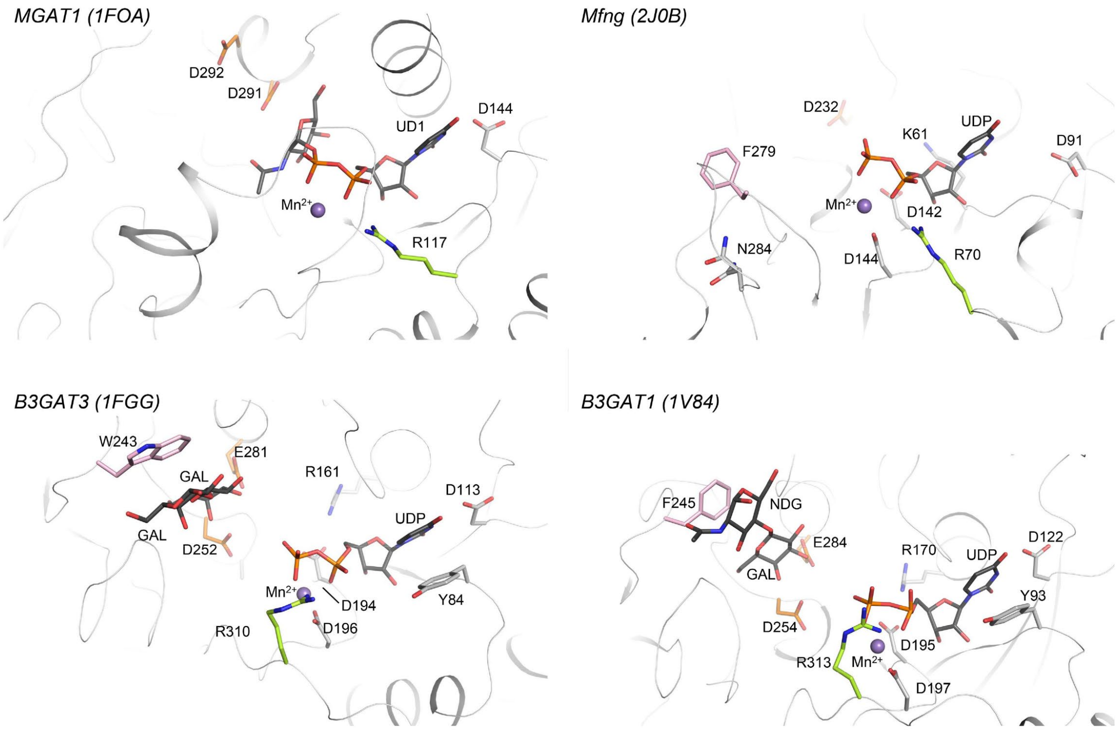

Figure S6: structural comparison between LH3/PLOD3 and other glycosyltranferases. The conserved aminoacids are shown as sticks; where present, Mn^{2+} cofactor and UDP-sugar are shown as purple sphere and black sticks respectively. Protein name and related PDB ID are indicated for each panel. Colour coding is as in Figure 2A and is maintained throughout the structures allowing to compare the LH3/PLOD3 critical residues with the other structures. For additional information on the proteins indicated in this panel refer to Table S2.

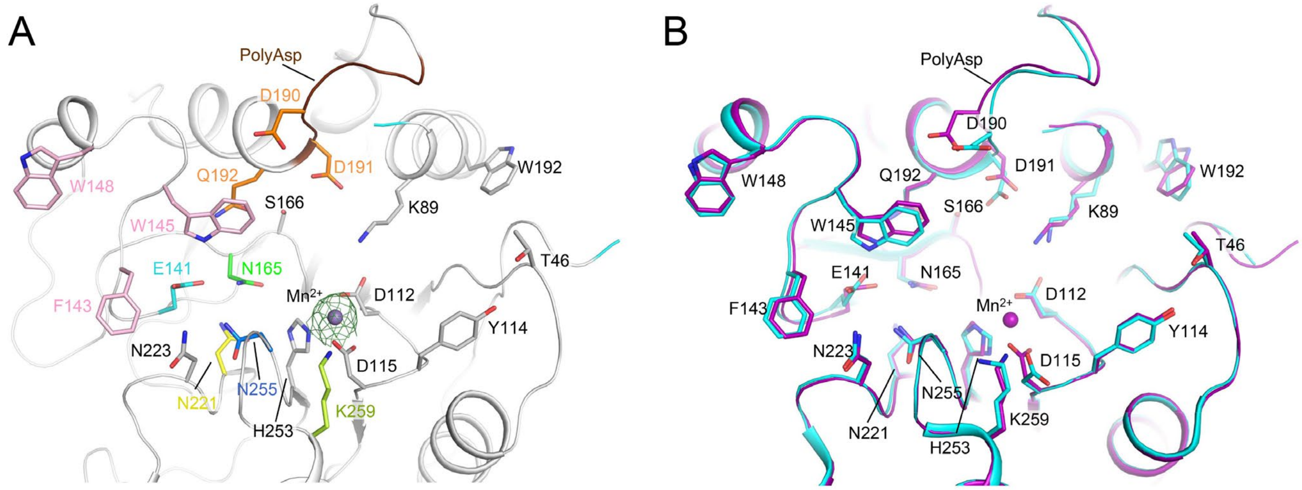

Figure S7: comparison of the GT domains of wild-type LH3/PLOD3 in ligand-bound and ligand-free states. (A) The structure of wild-type LH3/PLOD3 crystallized in presence of Mn^{2+} and UDP shows clear electron density for Mn^{2+} ($2F_o$-F_c omit electron density map shown as green mesh, contour level 2 σ), but unexpectedly no density is present for UDP. Residue highlight and colouring as in Figure 1A. (B) The comparative superposition of wild-type LH3/PLOD3 GT domain co-crystallized in presence of Mn^{2+} and UDP (purple), and without cofactors (cyan – PDB ID: 6FXK) shows no differences in the conformations of the side chains for the residues delimiting the Glc-T catalytic site.
